# Supplementary material for: FLAgellum Member 8 modulates extravascular distribution of African trypanosomes
Source: PLoS Pathog. 2023 Dec 21;19(12):e1011220. doi: 10.1371/journal.ppat.1011220 (PMC10769064; doi:10.1371/journal.ppat.1011220)
Supplement: Attachment — Submitted filename: Responses to reviewers V2.pdf [file ppat.1011220.s009.pdf]

Point-by-point response letter to reviewers' comments

We are happy to read that all reviewers appreciated our manuscript. We thank the editors as well as all the reviewers for their careful reading and their useful suggestions. We have addressed their comments in the point-by-point response letter hereafter. The corresponding modifications are highlighted in yellow in the main text.

Part I – Summary

Editors:

Provide some additional comment on heterogeneity among FLAM8KO clones, e.g. Fig 6A result that addback does not fully rescue. Incomplete rescue is not, per se, a major problem, as it is recognized that addback of a single allele combined with a complex phenotype and technologically challenging assay may explain the heterogeneity among clones. However, more comment on heterogeneity among KO clones, particularly vs addback in Fig 6A, and acknowledgment of the relatively noisy data in some cases (e.g. per reviewer 1 comments about Fig 3 and 4) needs to be made. Currently the results and Discussion sections tend to gloss over this.

Some more comments on these inter-clones and inter-experiments variations were added in the revised discussion.

Reviewer #3:

The authors have addressed my main concerns. Some of the proposed experiments/analysis were undertaken.
In my opinion, Figures 3D-E still appear to show that the phenotype of FLAM8 is different early and late in infection. But a more robust analysis using windows of 3-4 days would probably be more revealing than a daily statistical comparison.
Although the mechanism remains unknown, these findings open the door to understanding how T. brucei parasites extravasate into tissues.

Thank you for these positive comments.

Part II – Major Issues: Key Experiments Required for Acceptance

Reviewer #1:

While the revised manuscript has addressed some of the reviewer comments, I still have reservations about the reproducibility of the key data in Figure 3 and 4. The decline in the EV populations in the FLAM8 KO are fairly small, at least how they are depicted in Fig 3E.

Fig3E shows fold differences of EV populations at log scale, hence, the overall curve variations may not appear impressive at first sight. However, highly significant differences in EV populations were observed at different time points and indicated with asterisks.

It seems like the normalization method, such as the dissemination shown in Fig 3F (comparing time points 19-27), can greatly influence the magnitude and significance of the differences in parasite number, which makes it hard to know which is an accurate representation of the results.

In Fig3F, as well as in the other panels of Fig3 and 4, all values were compared between groups at each time point. This approach takes into account both the mean values and standard deviations. Therefore, although differences observed between days 19 and 27 may appear substantial on the graphical representation, they are actually not significant after careful quantitative analyses.

This is even more concerning in Figure 4, where many of the significant differences that were seen in Fig 3 don't appear to reproduce. I realize that in vivo infections come with a fair amount of heterogeneity, but it feels that we have two experiments here that are saying different things, with the interpretation heavily leaning on only one outcome. If the results in Fig 3 and Fig 4 are given equal weight, then the FLAM8 KO appears to impact EV dissemination early in the infection, but not during later stages.

As mentioned in the manuscript and as discussed in the previous answer, the experimental designs of these two experiments are different, with a higher time-resolution in experiment 1 (Fig 3) and a RT-qPCR endpoint target for experiment 2 (Fig 4).

The additional confounder here is that the qPCR data in Figs 4D-E appears to conflict with the data Fig 4B-C, showing fairly clear differences in parasite numbers (are they statistically significant?) during the late stages of the infection that are not captured with the whole animal imaging. This brings up the possibility that there are significant differences between WT and the FLAM8 KO EV dissemination, but at the expense of suggesting that the whole-animal imaging approach is not reliable/sensitive enough to pick up the differences in parasite numbers in this implementation. If this is the case, I don't know how to interpret the results in the paper.

As mentioned in the manuscript and as discussed in the previous answer, time-lapse live imaging of the whole organism and one-shot RT-qPCR on specific tissues are distinct and complementary approaches in space and time. In Fig 4, results obtained with these two methods can only be compared for the same time point, i.e. at the end of the experiment. Consequently, we don’t understand the reviewer comment.

Part III – Minor Issues: Editorial and Data Presentation Modifications

Reviewer #1: In Figure 5E, I find it strange to average the parasite levels for different organs, considering differences in size and what they can tolerate in terms of parasite burden. Is this the only way to get statistics for the organ parasite numbers?

This representation was actually requested by one reviewer at the first round of evaluation.
