# Supplementary material for: FLAgellum Member 8 modulates extravascular distribution of African trypanosomes
Source: PLoS Pathog. 2023 Dec 21;19(12):e1011220. doi: 10.1371/journal.ppat.1011220 (PMC10769064; doi:10.1371/journal.ppat.1011220)
Supplement: Attachment — Submitted filename: Responses to reviewers V2.docx [file ppat.1011220.s008.docx]

**Point-by-point response letter to reviewers’ comments**

We are happy to read that all reviewers appreciated our manuscript. We thank all the reviewers for their careful reading and their precise and useful suggestions. We have addressed their comments in the point-by-point response letter hereafter. The corresponding modifications are highlighted in yellow in the main text.

**Part I – Summary**

Reviewer #1:

The study provides important insights into the function of FLAgellar Member 8 (FLAM8) in Trypanosoma brucei infection by demonstrating its role in mammalian tissue colonization. While FLAM8 is not essential for parasite survival and growth in vitro or stumpy differentiation, its absence resulted in “impaired” dissemination in the extravascular compartment and decreased trans-endothelial migration. The authors propose that FLAM8 functions as a docking platform for Cyclic AMP response protein 3 (CARP3), which plays a role in signalling, and proposed that environmental sensing and/or signalling may play a role in extravasation.
Overall, this study sheds light on the mechanisms involved in the colonization of tissue-dwelling T. brucei parasites in mammalian hosts. There are a few important issues that should be addressed.

Thank you for these positive comments. See our detailed answers below.

Reviewer #2:

This study by Alvarez et al. investigates the function of the protein FLAM8 in the protozoan parasite Trypanosoma brucei. Recent work from several groups has shown that during the insect stage in the parasite’s life cycle, FLAM8 is part of a complex at the tip of the parasite flagellum that includes adenylyl cyclase and CARP3 and disruption of this complex was shown to prevent colonization of the tsetse fly salivary glands. Intriguingly, FLAM8 localisation changes to a more dispersed flagellar signal in the bloodstream form that dwells in the bloodstream and tissues of the mammalian host. Based on this difference in localization this study was conducted to discover if FLAM8 has a distinct function in this stage.

The results show convincingly that FLAM8 is not essential for the survival of bloodstream forms in vitro or in vivo. The initial strategy of depleting the transcript by RNAi is a valid approach but since under knockdown conditions the cells maintained 40% of the transcript levels compared to controls, this does not allow for strong conclusions. More compelling evidence that FLAM8 is dispensable under the conditions tested is provided by the knockout strategy that was chosen next, which was to replace one allele completely and the second allele partially. The justification for this strategy was that it facilitated construction of rescue lines by adding back the missing portion. The validation of gene loss/truncation was done at the level of DNA and absence of RNA and protein expression was also tested. Overall, reasonable evidence is given that a functional null mutant was achieved. By all measures that were applied these mutant cells behaved the same in vitro as the control cells.

In vivo, in a mouse infection model, these FLAM8 mutants were able to establish a bloodstream infection and they were also found in extravascular sites. Overall these data show that bloodstream forms lacking FLAM8 remain infective, and the data even shows that they can differentiate to the stumpy forms capable of colonizing tsetse flies. These conclusions are well supported by the data.

The authors then analyse in more detail the proportion of parasites in extravascular spaces. The stated conclusion is that “FLAM8-null parasites exhibit a significantly impaired dissemination in the extravascular compartment, that is restored by the addition of a single rescue copy of FLAM8.”

While some of the data is compatible with this claim, there are major questions as to the strength of this data.

Thank you for these overall positive comments. See our detailed answers below.

Reviewer #3:

The role of flagellum in Trypanosoma brucei motility is well documented, but its role in environment sensing remains poorly explored. In this study, the authors investigated the phenotype of pleomorphic parasites lacking Flagellar member 8 during infection of the mammalian host. The authors discovered that FLAM8 ko parasites are defective in disseminating within the mammalian host. In vitro experiments indicate that this defect could be due to a deficiency in crossing the endothelial vessel wall. FLAM8 interacts with a cyclic AMP response protein 3 (CARP3) and this protein is displaced from the cell surface in the absence of FLAM8 is. The authors speculate that the dissemination defect could be due to poor sensing of the environment and deficient cAMP signaling.

The most important novelty of this work is the identification of the first parasite protein that shows a defect in tissue colonization. The depletion of this flagellar protein has little fitness impact in vitro, providing important knowledge that the parasite flagellum is crucial for parasites to extravasate into tissues.

The paper is well written and with a clear story line. However, more convincing show that the FLAM8 ko defect is robust and differentiation- and motility-independent, three major points need to be addressed experimentally.

Thank you for these positive comments. See our detailed answers below.

**Part II – Major Issues: Key Experiments Required for Acceptance**

Reviewer #1:

1. The authors did a good job showing the variabilities that occur during T. brucei infection, especially by using three clones of the FLAM8 KO. However, there seems to be a fair amount of heterogeneity in the progression of infection that makes it hard to specifically say what the role of FLAM8 is during infection, and more directly what happens to the extravascular parasites, which is where the authors focus most of their work. In the first round of experiments, shown in Figure 3, there are significant differences in the EV parasites at day 7 onwards. In a repeat of this experiment, shown in Figure 4, it appears the EV parasites and dissemination measurements show that the FLAM8 KO achieves similar numbers after day 17. Considering the inconsistencies here, along with the smaller (but significant) differences seen at later stages of infection in the Fig 3 replicate, it seems that the simplest interpretation is that the FLAM8 KO is showing a delayed colonization of the extravascular compartments.

It is true that inter-individual heterogeneity is common during experimental *in vivo* infections. Here, we especially observe that KO group 2 at day 8 in Fig3 C and D shows lower mean EV populations and dissemination as compared to the two other groups, and that KO group 1 at day 5 in Fig4 B and C displays lower mean EV populations and dissemination as compared to the two other groups. Nevertheless, the overall dynamics in parasite populations in the 3 groups of mice infected with each of the 3 FLAM8 -/- sub-clones are following very comparable trends in both experiments. Both data sets show that FLAM8 KO are not delayed in colonizing the extravascular compartments, but their EV populations remain below the parental and rescue populations over most of the infection course as shown in the new Fig.3E. This is confirmed by qPCR analyses at the end of the experiment in Fig 4D-E.

The authors have used terms like “modulate, “contribute to the maintenance”, “involvement” to describe that FLAM8 is doing here, which is vague. The authors should clarify what they think is going on.

Yes, we are cautiously using these terms on purpose, as the full mechanisms involving FLAM8 are not completely known. Yet, the second part of the discussion is dedicated to proposing several hypotheses for some possible roles of FLAM8 in parasite extravasation. Note that another reviewer asked us to tone down this speculative part.

It would also be better to show the individual data points that reflect the parasite numbers in each mouse, rather than just an average with error bars.

Yes, we are now providing two new figures (FigS4 and FigS5) showing the individual data (IV, EV and dissemination) for all group of mice for both experiments.

2. Along the same lines, can the authors provide some explanation for why FLAM KO clone 2 shows similar transmigration rates to the FLAM KO rescue? That clone had the lowest rate of EV parasites, at least by Fig 4D and 4E. It would seem that if there is some heterogeneity in infective capacity of the specific pleomorphs FLAM8 KO clones, the clone that retains near-wild type transmigration capacity should be better at entering the extravascular spaces.

Yes, we have also noticed this, and we agree with the reviewer’s comment. This is difficult to explain. There is indeed likely some heterogeneity in the infective capacity of the specific pleomorphic FLAM8 KO clones, hence the importance of testing different clones.

3. The change in localization of FLAM8-CARP3 from the flagellar tip in PCFs to along the length of the flagellum in BSFs is very interesting, but there is nothing that supports the idea that this change in localization reflects a change in function. Both complexes are flagellar- there could be other reasons for why FLAM8-CARP3 change localization within the flagellum. The authors should show that that the change in localization leads to a change in function or tone down their interpretations at the end of the discussion (lines 353-372).

We agree and we have toned down our interpretations regarding the possible correlation between the change in localization and a change in function.

Reviewer #2:

1. Based on the data in Figures 3C and 3D the result is reported that dissemination / extravascular parasite numbers are lower for the mutants. Significant differences are noted for days 5-12 and again from day 19. If dissemination was impaired, what plausible explanation is there for the lack of a difference in the week between days 12-19?

The overall parasite distribution in each compartment evolves differently and in a dynamic manner over the course of an infection. Yet, within each compartment, the parasite distribution is likely not homogenous (different vascular sub-compartments, different organs and tissues). Here, this is exemplified between days 12 and 19, when comparing the total EV parasite populations (Fig3C) and their spatial distributions (Fig3A and 3D). This is also observed by qPCR analyses at the end of the experiment in Fig 4D-E. In total, the overall dynamics in parasite populations in the 3 groups of mice infected with each of the 3 FLAM8 KO sub-clones are following very comparable trends in both experiments, with a limited and variable EV distribution as compared to the parental and rescue strains.

The variation between the subclones is quite striking, and unexplained. The experiment was once repeated (Fig 4) and again, there was great variation between the clones in all tested conditions. The IV parasite data in Fig 4 A is particularly noisy, and different from the first experiment (Fig 3A).

It is true that inter-individual heterogeneity is common during experimental *in vivo* infections. Here, we indeed observed some variations in the dynamics of IV parasites between subgroups of mice infected with the different KO subclones, especially for group 3 when compared to the two other groups after day 9. However, we do not think these variations are striking as these IV parasite population values were dynamics in the same range and because the overall dynamics in EV parasite populations in the 3 groups of mice infected with each of the 3 FLAM8 KO sub-clones are following very comparable trends in both experiments.

Given this variation, infecting only three mice with each cell line seems like a small number. This issue needs to be addressed e.g. by clarifying what calculations were done to assess that this was the appropriate number.

Yes, the sample size calculation is now addressed in the material and method section. Note that all the 18 mice infected with FLAM8-/- trypanosomes showed very comparable levels and dynamics of EV parasite populations.

In Fig 4B and C initial differences are seen but the mutants all reach the same end point as the parental cell line. A much clearer difference is detected in Fig 4D where parasite burdens in isolated organs were quantified on day 24. However, this seems inconsistent with the data in Figs 4B and C that show NO difference on day 24.

As previously discussed, within each compartment, the parasite distribution is likely not homogenous (different vascular sub-compartments, different organs and tissues). On day 24, all three KO mutants present a lower, yet not statistically significant, dissemination as compared to the parental control (Fig4C). This parameter mostly reflects the dissemination of parasites in the skin. In Fig4D, the differential number of parasites in the skin of null mutants as compared to the parental and rescue strains confirms this trend. However, the differential numbers of parasites between strains in other and deeper organs don’t impact the dissemination parameter as much as dermal parasites.

2. In a transmigration assay with HUVEC cells, a difference is reported in the transmigration between FLAM8 knockouts and parentals. The rescue cell line has an “intermediate” phenotype. The fact that it has only one allele is given as a possible explanation. However, the localization images in Fig 2 show no difference in FLAM8 signal between parental and rescue and no quantification is shown, leaving the question open how gene dosage relates to protein abundance and consequently phenotype.

Due to the large size of FLAM8 (3,075 amino acids), and despite multiple attempts in different conditions, we were unable to quantify the protein level by Western Blot. However, as in Calvo *et al.* Cell Microbiol 2021, we performed a comparative immunofluorescence analysis of the FLAM8 fluorescent signal intensities measured in ROI drawn along parasite flagella. The total fluorescence intensities were compared between strains and plotted in a new Fig2B. As expected, an intermediate level of FLAM8 expression was observed in the rescue strain, most probably due to the presence of a single allele and explaining the only partial rescue phenotype observed with this strain.

Moreover, the observation that the partial rescue did worse in the transmigration than one of the KOs and only slightly better than the other two KOs raises the question whether this measurement truly shows a rescue effect.

Due to the presence of a single allele of FLAM8, the rescue cell line has only an “intermediate” rescue phenotype in all experiments performed. Regarding the transwell data, we were rather surprised by the transmigration level of clone 2 that is difficult to explain. There is indeed likely some heterogeneity in the infective capacity of the specific pleomorphs FLAM8 KO clones, hence the importance of testing different clones.

It is also unclear which KO was the parent of the rescue. Considering the variation between KO clones, paired comparisons of KO clones with rescue lines derived from said KO are needed to allow for any conclusions.

The FLAM8 KO clone used to generate the rescue cell line is #1. This is now mentioned in the Material and methods section.

3. The final experiment (Fig 6C) shows CARP3 was no longer detected in FLAM8 KO cells by immunofluorescence and it was detected at reduced levels in the rescue line. Images of only one cell each is presented as evidence. This should be backed up by a more quantitative measure, either from fluorescence measurements on a larger cell population or alternatively comparing protein levels e.g. on a Western blot. correlating the level of FLAM8 (using the antibody as in Fig 2) with the levels of CARP3 would also be important to make the link between the FLAM8 depletion phenotype and possible involvement of CARP3/cAMP signaling.

To answer this pertinent suggestion, as in Calvo *et al.* Cell Microbiol 2021, we performed a comparative immunofluorescence analysis of the CARP3 fluorescent signal intensities measured in ROI drawn along the flagellum in the same conditions as in Fig6C and Fig2A. The total fluorescence intensity levels were compared between strains and plotted in a new Fig6D. As expected, the CARP3 expression dynamics was comparable to that of FLAM8 in all strains. Interestingly, the loss of CARP3 in the 3 FLAM8 KO clones was mostly observed in the proximal region of the flagella in most cells, here again demonstrating the presence of at least two distinct pools of CARP3, one being independent of FLAM8.

The claim that the phenotype is “possibly due to cAMP signaling impairments” is at this point speculative. There is no evidence in this paper that the defect reported for bloodstream forms has anything to do with cAMP.

This is true and we removed this sentence from the abstract.

Minor point: It is notable that Fig 6C shows not only lack of CARP3 signal in the flagellum but complete absence of any signal. Assuming the complete absence of CARP3 signal shown in Fig 6C is representative of the FLAM8 KO population, how is this interpreted? Do the authors conclude that absence of FLAM8 not only prevents CARP3 from localizing to the flagellum, but also leads to the degradation of CARP3 protein?

Cell shown in the Fig6C were fixed in methanol for 30 minutes following our optimal protocol for imaging FLAM8. Hence, soluble proteins in the cytosol or in the flagellum matrix are unlikely to be detectable in these conditions. Interestingly, we found that the loss of CARP3 in the 3 FLAM8 KO clones was mostly observed in the proximal region of the flagella, here again demonstrating the presence of at least two distinct pools of CARP3. This doesn’t exclude an additional soluble pool of CARP3.

Overall my view is that the claims about a dissemination defect due to reduced extravasation are not strongly supported by the data. The effect is modest and temporary, in vivo. The number of biological repeats is small and the variation between mutant cell lines is large, both in vivo dissemination measures and in the transwell assay. The rescue is partial and some of the data is inconsistent as noted above.

The observed phenotype is indeed not Boolean nor massive, and it evolves over the infection course. However, it is significantly associated to the loss of FLAM8 according to multiple parameters from multiple experiments in multiple replicates with appropriate controls. A total of 18 mice were infected with a FLAM8 KO mutant with comparable profiles for all parameters (excepted IV parasites in Fig 4C). Most aspects have been explained / detailed in our point-by-point answers to the reviewers.

The availability of the in vitro transmigration assay offers the opportunity to dissect the observed phenomena more fully. For example, the model proposed by the authors would predict that depletion of CARP3 would lead to a similar transmigration defect. This would be an important experiment.

The CARP3 KO strains used in BACHMAIER S nat comm 2022 are matrix-dependent, which is not compatible with our transwell protocol. We have tried the proposed experiment, with multiple adjustments, yet unfortunately without success, as the internal technical controls at baseline remained negative (no transmigration of parental cells in liquid medium).

Reviewer #3:

The authors concluded that in FLAM8 knockout does not affect parasite motility in vitro, by measuring parasite speed and linearity in the presence of 0,5% methylcellulose. However, previous work by the Engstler lab (10.1371/journal.ppat.1005448) has shown that the type of motility displayed by T. brucei is highly dependent on the viscosity of the medium, which could reflect the viscosity of intra- and extravascular spaces. Therefore, it is crucial to demonstrate that FMAL8 knockout parasites have no motility defect at different concentrations of methylcellulose. Without these experiments or intravital imagining, the authors cannot exclude the possibility that FLAM8 knockout parasites have a motility defect in vivo.

We thank the reviewer for this suggestion and agree that this additional control is relevant indeed. Hence, we repeated the experiment shown in Fig.2D-E, but in the presence of 1.1% methylcellulose. The new data were added as Fig.2G-H. As expected, FLAM8 mutants did not show any motility defect in high viscosity medium (1.1% methylcellulose).

The phenotype of parasite distribution in FLAM8 knockout in mice (Figures 3B-C) is not apparent due to many lines and many error bars that impede a clear interpretation of the graphs. Compared to wildtype parasites, FLAM knockout parasites appear to have lower numbers inside and outside vessels during the first week of infection. In later stages of the infection, FLAM8 knockout parasites appear to have higher numbers inside vessels and lower numbers outside vessels. It would be helpful if the authors could quantify the mean fold-difference between wildtype/knockout/AB cell lines at the different stages of infection and identify which differences are statistically significant. Moreover, it would be interesting to explain the differences in phenotype in early and later stages of infection.

We are now providing new Fig3D-E presenting the fold change dynamics of IV and EV populations of all mutant strains normalized to the parental populations. It shows that FLAM8 KO are not delayed in colonizing the extravascular compartments at the beginning of the experiment. However, their EV populations remain significantly below the levels of the parental population over most of the infection course, while their IV populations remain higher than the parental strain.

Assessing defects in differentiation *in vivo* is not trivial. The authors used PAD1 mRNA levels as a proxy for measuring the proportion of stumpy forms. It would be beneficial for the authors to perform additional assays such as cell cycle analysis by IFA (counting N and K) in tissue sections and/or morphology.

We agree that assessing defects in differentiation *in vivo* is indeed not trivial. Cell cycle analysis and morphometry in tissue sections would be very time-consuming and with an output of limited quality since trypanosomes do not necessarily entirely lie down on histological section plans.

Additionally, it is important to consider the total parasite density in each organ and see if it is correlated with proportion of stumpy forms. Drawing conclusions about differences in the proportion of stumpy forms across organs (Figure 5F) based on just one time point of infection should be avoided. Figure 5G is adequate and compelling in demonstrating that FLAM8 ko does not appear to affect differentiation in vivo.

We agree that Fig.5F was not clear enough and removed it.

The connection between cAMP signaling and FLAM8 phenotype is solely based on changes in CARP3 localization, which is a weak link. Therefore, the speculation that FLAM8 phenotype may be due to sensing functions should be toned down.

Yes, we toned down the discussion in the revised version.

**Part III – Minor Issues: Editorial and Data Presentation Modifications**

Reviewer #1:

1. Line 110: The authors may consider adding "life" to the sentence to clarify the protein's differential localization in different life cycle forms rather than cell cycle stages

Added

2. Fig 5D: It would be helpful if the authors could explain how the ePCFs were characterized, whether based on morphology or the expression of a molecular marker.

No specific marker was used, and these stages were referred to as ePCF only because they were collected 24h after induction. We clarified this by simply using PCF in the revised version.

3. Fig 6B: Could the authors comment on the variation observed in the different KO lines?

The reason why FLAM8 KO clone 2 cells present a higher transmigration efficiency as compared to the two other clones is neither obvious for us, however all three mutants have a significantly lower transmigration efficiency as compared to their parental strain.

4. Fig 4 D-E: Could the authors explain the Y-axis in the figure legend to facilitate the comprehension of the graph by readers?

This is now more detailed in the legend.

5. It would be interesting to investigate if expressing FLAM8 ectopically leads to more parasites migrating to the extravascular environment.

Yes, it would be interesting indeed, although we could hypothesize that the endogenous amount of FLAM8 partners could limit a possible effect.

6. For the IF on CARP3 in Fig 6- are they using the same exposure/lookups? The CARP3 levels in the WT looks a lot lower than the add back. If there is a difference it would merit comment.

Fig6C shows a panel of fluorescent pictures that were all normalized to the same intensity display settings for direct visual comparison of CARP3 expression levels in all strains. A new picture panel is now provided in Fig6C together with a fluorescence quantification comparing all strains in Fig6D. As expected, CARP3 was detected along the entire flagellum length in parental mammalian forms, but not in Δ*FLAM8* parasites where the fluorescent signal was weaker and restricted to the distal part of the flagellum (Fig. 6C-D). This demonstrates the presence of at least two distinct pools of CARP3, one being independent of FLAM8 for its localization to the anterior part of the flagellum. The presence of a *FLAM8* rescue copy in Δ*FLAM8* parasites restored the detection of CARP3 along the entire flagellum, yet in lower amounts than in parental cells (Fig. 6C-D).

7. Figure 3B would benefit from a change to the Y-axis, perhaps with a 10^8 maximum, to spread out the data more. As currently plotted, it is very difficult to interpret day 5 to day 13.

Using the same scale for both IV and EV parasite populations highlights the fact that EV parasite population is by far the largest (higher by 2 logs). Consequently, variations of the EV populations might more significantly impact the infection process, and this is why we mostly focus on the EV population fluctuations.

8. Line 409: The website that the authors list for RNAit is no longer functional. There is a new one based out of Dundee for RNAit2.

Thank you for the update! This was modified.

Reviewer #3:

To understand how intravascular parasites (Fig 1E) were counted, the reader has to check the Materials and methods section. Given that extravascular parasites (Fig 1F) are determined by subtracting this value from the number of parasites measured by total bioluminescence (Fig 1D), it would be important that the Results section or the legend of Figure 1 explains that intravascular parasites were counted from a tail bleed using a cytometer.

This precision was added.

Is Figure 4A a replicate of Figure 3B? It sems completely different. How do authors explain such differences between experiments and what does it say about the FLAM8 phenotype?

These two figures show two distinct biological replicates. It is true that inter-individual heterogeneity is common during experimental *in vivo* infections. Here, we especially observe that KO group 2 at day 8 in Fig3 C and D shows lower mean EV populations and dissemination as compared to the two other groups, and that KO group 1 at day 5 in Fig4 B and C displays lower mean EV populations and dissemination as compared to the two other groups. Nevertheless, the overall dynamics in parasite populations in the 3 groups of mice infected with each of the 3 FLAM8 -/- subclones are following very comparable trends in both experiments. We are now providing new FigS4 and S5 presenting the dynamics of IV populations, EV populations and dissemination for each individual mouse for both experiments.

The way in which the parasite densities are displayed in the graphs of Figure 4D-E is not very user-friendly. It would be beneficial for readers to not only see the delta, but also be aware of the total number of parasites for each organ and the ratio delta/total, which indicates the proportion of parasite population is missing in a given organ upon FLAM8 knockout. Currently, the way the data is plotted makes it difficult to comprehend the relative effect size. Additionally, error bars are missing in panel D, and legend of Y axis should be more precise in explaining what Delta represents. Finally, the figure legend should indicate the day of infection when the organs were collected.

The legends of Fig4D and 4E were modified to better explain the calculation method and the data displayed. For clarity purposes, the format of Fig4D was kept but a new Fig S6 is now provided to show individual Delta no. of EV parasites for each organ from each mouse dissected at D24.

The way the proportion of stumpy forms is displayed in Figure 5F-G is confusing. A value of 3 to 9 in Delta is not intuitive.

Fig5F was removed as it was not clear enough. The previous Fig5G (now Fig. 5E) simply demonstrates that FLAM8 KO does not appear to affect differentiation *in vivo*.

The title of the paper is confusing and long. Would the authors consider the alternative title “FLAgellum Member 8 modulates extravascular distribution of African trypanosomes.”

Thank you for the suggestion. The title was modified accordingly.
